# Inhibitory Receptors Are Expressed by *Trypanosoma cruzi*-Specific Effector T Cells and in Hearts of Subjects with Chronic Chagas Disease

**DOI:** 10.1371/journal.pone.0035966

**Published:** 2012-05-04

**Authors:** Rafael J. Argüello, María C. Albareda, María G. Alvarez, Graciela Bertocchi, Alejandro H. Armenti, Carlos Vigliano, Patricia C. Meckert, Rick L. Tarleton, Susana A. Laucella

**Affiliations:** 1 Instituto Nacional de Parasitología “Dr. Mario Fatala Chaben”, Buenos Aires, Argentina; 2 Chagas Disease Section, Hospital Interzonal General de Agudos “Eva Perón, Provincia de Buenos Aires, Argentina; 3 Departamento de Patología, Hospital Universitario Fundación Favaloro, Buenos Aires, Argentina; 4 Center for Tropical and Emerging Global Diseases, University of Georgia, Athens, Georgia, United States of America; Escola Paulista de Medicina - UNIFESP, Brazil

## Abstract

We had formerly demonstrated that subjects chronically infected with *Trypanosoma cruzi* show impaired T cell responses closely linked with a process of T cell exhaustion. Recently, the expression of several inhibitory receptors has been associated with T cell dysfunction and exhaustion. In this study, we have examined the expression of the cytotoxic T lymphocyte antigen 4 (CTLA-4) and the leukocyte immunoglobulin like receptor 1 (LIR-1) by peripheral *T. cruzi* antigen-responsive IFN-gamma (IFN-γ)-producing and total T cells from chronically *T. cruzi*-infected subjects with different clinical forms of the disease. CTAL-4 expression was also evaluated in heart tissue sections from subjects with severe myocarditis. The majority of IFN-γ-producing CD4^+^ T cells responsive to a parasite lysate preparation were found to express CTLA-4 but considerably lower frequencies express LIR-1, irrespective of the clinical status of the donor. Conversely, few IFN-γ-producing T cells responsive to tetanus and diphtheria toxoids expressed CTLA-4 and LIR-1. Polyclonal stimulation with anti-CD3 antibodies induced higher frequencies of CD4^+^CTAL-4^+^ T cells in patients with severe heart disease than in asymptomatic subjects. Ligation of CTLA-4 and LIR-1 with their agonistic antibodies, in vitro, reduces IFN-γ production. Conversely, CTLA-4 blockade did not improved IFN-γ production in response to *T. cruzi* antigens. Subjects with chronic *T. cruzi* infection had increased numbers of CD4^+^LIR-1^+^ among total peripheral blood mononuclear cells, relative to uninfected individuals and these numbers decreased after treatment with benznidazole. CTLA-4 was also expressed by CD3^+^ T lymphocytes infiltrating heart tissues from chronically infected subjects with severe myocarditis. These findings support the conclusion that persistent infection with *T. cruzi* leads to the upregulation of inhibitory receptors which could alter parasite specific T cell responses in the chronic phase of Chagas disease.

## Introduction

Chagas disease, caused by *Trypanosoma cruzi* infection, is the most frequent cause of infectious cardiomyopathy in the world, with approximately 4 million individuals presenting heart disease [1]. CD4^+^ and CD8^+^ T responses are involved in the control of the acute *Trypanosoma cruzi* infection and keep the parasite burden under control during the chronic phase of the infection. Phenotypic analysis of the inflammatory lesions in the heart of chronically infected subjects showed a predominance of CD8^+^ T cells, a smaller proportion of CD4^+^ T cells, as well as the presence of B lymphocytes, plasmatic cells, macrophages, eosinophils and mastocytes [2], [3].

A defining feature of memory T cells generated after acute infections is the long-term antigen-independent persistence mediated by homeostatic turnover, as demonstrated in viral infections [4], [5]. In contrast, during chronic infections, differentiation of antigen-specific T cells may occur differently, where specific antigen is essential for maintenance of antigen-specific T cells [6]–[9]. T cells initially acquire effector functions but gradually become less functional as the infection progresses. This loss of function, known as exhaustion, is hierarchical, with the proliferative potential and production of interleukin 2 (IL-2) lost early, followed by the ability to make tumor necrosis factor alpha, while IFN-gamma (IFN-γ) production is most resistant to functional exhaustion [5], [10].

A large number of surface markers have been used to define phenotypically distinct populations of CD4^+^ and CD8^+^ T cells at different stages of differentiation [11], [12]. The expression of CD45RA, CD62L, CD127, CD28 and CCR7 on antigen-specific T cells defines cell populations at early stages of differentiation (i.e. naïve and central memory T cells), while the lack of expression of CD45RA, CD62L, CD127, CD28 and CCR7 defines cell populations at late stages of differentiation (i.e. effector memory T cells). Besides, the expression of CD57 and CD45RA linked to a high expression of the cytotoxic factor perforin A, in the absence of the other phenotypic markers, has been associated with cell populations at the terminal stage of differentiation (i.e. terminally differentiated effector T cells) [11], [12].

Exhausted T cells display phenotypic markers that are typically associated with effector/effector memory T cell populations [10], [13] and display high levels of inhibitory receptors [14]–[18]. Among these inhibitory molecules are the cytotoxic T lymphocyte antigen 4 (CTLA-4/CD152) and the leukocyte immunoglobulin like receptor 1 (LIR-1/CD85j). CTLA-4 is upregulated on activated T cells but it is also involved in regulatory T cell functions of regulatory T cells [19], [20]. Like the related molecule CD28, CTLA-4 binds to B7.1 (CD80) and B7.2 (CD86), although with significant higher avidity. CTLA-4 reduces T cell activation by modulating the threshold of signals needed for T-cell cytokine production and proliferation [21]–[23]. Furthermore, CTLA-4 ligation can lead to negative effects on the regulation of cell cycle and inhibits the transcription factors nuclear factor κB, nuclear factor of activated T cells and activator protein 1. CTLA-4 has also been implicated in the upregulation of FoxP3 expression [23]. LIR-1 is one of the killer-cell immunoglobulin-like receptors present on the cell surface of a variety of immune cells, including T lymphocytes [24], [25], and its ligation with classical class I (HLA-A, B, C) and non-classical (HLA-E, F, G) MHC proteins leads to inhibitory signals to effector cells [26], [27]. It has been demonstrated that the expression of LIR-1 increases during differentiation of virus-specific CD8^+^ effector T cells [15].

We have previously shown that chronically infected subjects with no or mild clinical disease have a significantly higher frequency of interferon-gamma (IFN-γ) producing T cells specific for *T. cruzi* than do individuals with more severe disease [28]–[30], with single IFN-γ-producing T cells as the predominant functional profile [31]. Subjects with severe chagasic heart disease also display an increased frequency of fully differentiated total CD4^+^ [29] and CD8^+^ [30] T cells and high rates of T cell apoptosis, leading us to propose that long-term parasite persistence might drive the immune system to exhaustion [6].

Herein, we have examined the expression of CTLA-4 and LIR-1 by IFN-γ-producing CD4^+^ T cells in response to *T. cruzi* antigens in relation to the magnitude of cytokine production and disease severity. CTLA-4 and LIR-1 expression was also assessed on the overall T cell compartment and in heart specimens from chronically *T. cruzi*-infected subjects. Our results show that circulating IFN-γ producing CD4^+^ T cells from chronically *T. cruzi*-infected subjects display increased expression of CTLA-4 and/or LIR-1 in response to parasite antigens. CTLA-4 expression is also increased in inflammatory heart lesions from chronically infected subjects with intense myocarditis.

## Methods

### Selection of study population

*T. cruzi*-infected adults (n = 87) volunteers aged 35 to 68 were recruited at the Chagas disease Section of Hospital Interzonal General de Agudos “Eva Perón”, Buenos Aires, Argentina. *T. cruzi* infection was determined by indirect immunofluorescence assay, hemagglutination, and ELISA techniques [32]. Chronically infected subjects were evaluated clinically and stratified according to the Kuschnir grading system [33]. Group 0 (G0, n = 48; mean age = 50 y, range = 35–67) included seropositive individuals exhibiting a normal electrocardiography (ECG), and a normal chest-X ray; group 1 (G1, n = 10; mean age = 48 y, range = 36–56) seropositive patients had a normal chest-x ray but abnormalities in the ECG; group 2 (G2, n = 12; mean age = 51 y, range = 42–64) seropositive patients had ECG abnormalities and heart enlargement as determined by chest X-ray and group 3 (G3, n = 17; mean age = 55 y, range = 46–68) seropositive patients had ECG abnormalities, heart enlargement and clinical or radiologic evidence of heart failure. Eighteen subjects in the G0 group were treated with benznidazole as previously described [34], and followed for 12 to 50 months. Uninfected subjects comprised seronegative individuals from non endemic areas (SN non endemic, n = 13; mean age = 47 y, range = 41–55) and seronegative subjects from endemic areas (SN endemic, n = 7; mean age = 49 y, range = 39–58). Mean ages were not significant different among the subject groups evaluated. Heart tissue sections (i.e. heart explants) from either chronically *T. cruzi* infected subjects with severe cardiomyopathy (G3 group), patients with idiopathic dilated cardiomyopathy or subjects suffering from giant cell cardiomyopathy, who had undergone heart transplantation, were assessed for the expression of CD3, CTLA-4 and CD57 as described below. This protocol was approved by the Institutional Review Boards of the University of Georgia, and the Hospital “Eva Perón”. Signed informed consent was obtained from all individuals prior to inclusion in the study.

### Collection of PBMC

Peripheral blood mononuclear cells (PBMCs) were isolated by density gradient centrifugation on Ficoll-hypaque (Amersham, Sweden) and were cryopreserved for later analysis.

### Flow cytometry and intracellular cytokine staining assays

To assess the expression of CTLA-4 and LIR-1 by IFN-γ^+^ T cells, 2×10^6^ PBMCs were incubated with 15 µg/ml of an amastigote lysate preparation [29], 2.5 IU/ml of tetanus and diphtheria toxoids (TETADIF, BulBio, Bulgaria), 5 µg/ml of anti-CD3 antibodies (BD, USA) or media alone for 18 h, with the addition of 10 µg/ml brefeldin A (Sigma, USA) for the last five hours of incubation, as previously described [29], [30]. The cells were then stained with anti-CD4 (FITC) and anti-LIR-1 (PE-Cy5) monoclonal antibodies (BD, USA) followed by fixation and permeabilization for the intracellular staining with anti-IFN-γ (APC) and anti-CTLA-4 (PE) antibodies (BD, USA). CTLA-4 and LIR-1 expression was quantified in cytokine-producing T cells. IFN-γ responses were considered positive if they were at least three times the value of the unstimulated control.

For phenotyping of the total CD4^+^ and CD8^+^ T cell populations, PBMCs were stained with anti-CD8 (APC), anti-CD4 (FITC) and anti-LIR-1 (PE-Cy5) followed by fixation and permeabilization with cytofix/cytoperm kit (BD, USA) and staining with anti-CTLA-4 (PE). Data were acquired on a FACS Calibur cytometer (BD, USA) and analyzed with CellQuest software (BD, USA). Typically, 500.000 events were collected per sample.

For the characterization of LIR-1^+^ T cells, PBMCs were stained with the following combinations of monoclonal antibodies: CD4 or CD8 (APC), CD45RA (FITC), CD62L (PE) and LIR-1 (PE-Cy5), all from BD, USA; CD4 or CD8 (APC) (BD, USA), LIR-1 (PE-Cy5) (BD, USA), Perforin A (FITC), all from BD USA and CD57 (FITC) (Biolegend, USA); CD4 or CD8 (APC-Cy7) (Biolegend, USA), LIR-1 (PE-Cy5) (BD, USA), CD27 (PerCP) (BD, USA), CD28 (Pacific Blue) (eBioscience, USA) and CCR7 (PEcy7) (BD, USA). At least 600.000 events were acquired on a CyAn (DakoCytomation, Ft Collins, CO, USA) and further analyzed with Flowjo version 4.2 (Tree Star, San Carlos, CA, USA) software.

A cut off value for CD4^+^LIR-1^+^ T cells was set as the mean percentage ±2 SD from uninfected subjects. Changes in the levels of CD4^+^LIR-1^+^ T cells following treatment with benznidazole were considered significant when the post treatment/baseline differences were greater than the mean minus 2 standard deviation of post enrollment/baseline differences in 12 untreated chronically infected subjects.

### IFN-γ ELISPOT assays with cross-linking or blocking of CTLA-4 and LIR-1

The number of IFN-γ-secreting T cells in the presence of a plate bound (cross-linking) isotype control antibody, anti-CTLA-4 or anti-LIR-1 monoclonal antibodies (R&D Systems, USA) was determined by ex vivo ELISPOT using a commercial kit (ELISPOT Human IFN-γ Set; BD, USA), as described elsewhere [28], [31]. Briefly, nitrocellulose plates were coated with 100 µl of monoclonal mouse anti-human IFN-γ diluted in PBS (5 µg/ml) with the addition of anti-CTLA-4 monoclonal antibody (5 µg/ml clone BNI3, R&D Systems, USA), anti-LIR-1 (5 µg/ml clone GHI/75, R&D Systems, USA) or isotype control antibody (IgG2b 5 µg/ml, R&D Systems, USA) and incubated overnight at 4°C. Wells were then washed with PBS and incubated with complete RPMI for 2 hours. Cryopreserved PBMCs were seeded in duplicate wells, at a concentration of 4×10^5^ cells/well, and were stimulated with an amastigote lysate preparation (10 µg/ml) or media alone. For positive control, PBMCs were stimulated with 20 ng/mL Phorbol 12- Miristate 13-Acetate (Sigma, USA) plus 500 ng/ml ionomycin (Sigma, USA). After incubation for 16–20 h at 37°C in a 5% CO2 environment, cells were removed from plates and spots developed according to manufacturer instructions. Spot forming cells (SFCs) were automatically enumerated using CTL-ImmunoSpot S5 Core analyzer. Responses were considered as positive if a minimum of 25 SFCs/1×10^6^ PBMCs were present per well, and additionally, this number was at least twice the value of wells with media alone [31]. *T. cruzi*-specific responses were calculated by subtracting the number of SFCs from wells containing media alone from the *T. cruzi* lysate-stimulated spot count.

For blocking experiments, nitrocellulose plates were coated with monoclonal mouse anti-human IFN-γ alone. PBMCs were incubated with *T. cruzi* lysate (10 µg/ml) or media alone in the presence of either isotype control (50 µg/ml) or anti-CTLA-4 (50 µg/ml) antibodies for 16–20 hs. Afterwards, the SFCs were developed as described above.

### Tissues and Immunohistochemistry

Heart tissue sections (i.e. heart explants) from chronically *T. cruzi* infected subjects with severe cardiomyopathy (G3 group, n = 8, 4 men; mean age ± SD, 51.4±7.3 y; range 42–61 y) who had undergone heart transplantation were assessed for the expression of CTLA-4 (R&D Systems, USA) and CD57 (BD biosciences, USA) as described below. Heart explants from patients with giant cell myocarditis (n = 2, males, 28 and 47 y) and idiopathic dilated cardiomyopathy (n = 1, male, 45 y) were employed as controls. All patients were admitted at Hospital Universitario Fundación Favaloro, Buenos Aires, Argentina, to undergo orthotopic heart transplantation. Human tonsils and lymph node tissues from the Tissue Bank of the Pathology Lab were employed as positive controls of CTLA-4 [35] and CD57 [36] staining.

Explanted hearts were fixed for 72 h in 10% phosphate-buffered formaldehyde and transmural sections at the apex, atrium and of the whole circumference of the left and right ventricle at a plane equidistant from the base to the apex were collected and embedded in paraffin [37]. A 5-mm-thick section from each region was stained with hematoxylin and eosin and Masson's trichrome stain. The number of mononuclear cells was determined on each one out of 10 fields examined. The myocarditis was diagnosed if myocyte necrosis or degeneration, or both, associated with an inflammatory infiltrate adjacent to the degenerating or necrotic myocytes, could be demonstrated, according to the Dallas criteria [38]. The amount of inflammatory infiltrate was semi quantified as mild, moderate, or severe, and its distribution characterized as focal, confluent or diffuse. CTLA-4 and CD57 expression was evaluated by immunohistochemistry, as previously described [35].

Images were acquired with a digital camera (AxioCam Zeiss, USA) and analyzed with a digital analysis software from ImageJ software (NIH, USA). Ten representative fields of myocarditis were examined at 400× magnification and the number of CTLA-4^+^ and CD57^+^ positive cells out of total inflammatory mononuclear cells was determined.

Double labeling analysis on selected tissue sections derived from chronic Chagas disease patients showing high degree of inflammation were carried out by immunofluorescence by staining with anti-CTLA-4 (goat polyclonal antibodies, RD Systems, USA) and anti-CD3 (mouse monoclonal antibody, Santa Cruz, USA) antibodies. The secondary detection system was Alexa fluor 594 labeled anti-mouse immunoglobulins (Donkey anti-mouse) (Invitrogen, USA) and biotynilated anti-goat (rabbit polyclonal antibodies) ( Biogenex, Freemont, CA USA) followed by avidin-conjugated Fluorescein (Vector, Burlingame CA, USA). Nuclei staining were performed with ready to use mounting medium for fluorescence with diamidino-2-phenylindole (DAPI) (Vectashield, Vector, Burlingame, CA, USA). All reagents dilutions were used according to manufacturer data sheet instructions. Observations were made with a 100 W ultraviolet lamp and images were acquired with an AXIOCAM camera (Carl Zeiss AG, Oberkochen, Germany).

### Statistics

Kruskal-Wallis test and Dunn's post-test were used to compare differences between subject groups. One-way ANOVA with post test for lineal trend was used for trend analysis. Student's *t*-test was applied to analyze the quantitative differences between experimental and isotype control wells in crosslinking and blocking assays, as well to compare the percentages of CD45RA^+^/^−^CD62L^+^/^−^LIR-1^+^ cells between CD4^+^ and CD8^+^ T cells. Differences were considered to be statistically significant at *P*<0.05.

## Results

### *T. cruzi*-antigen-responsive IFN-γ-producing CD4^+^ T cells express CTLA-4 and LIR-1

**Figure 1:**
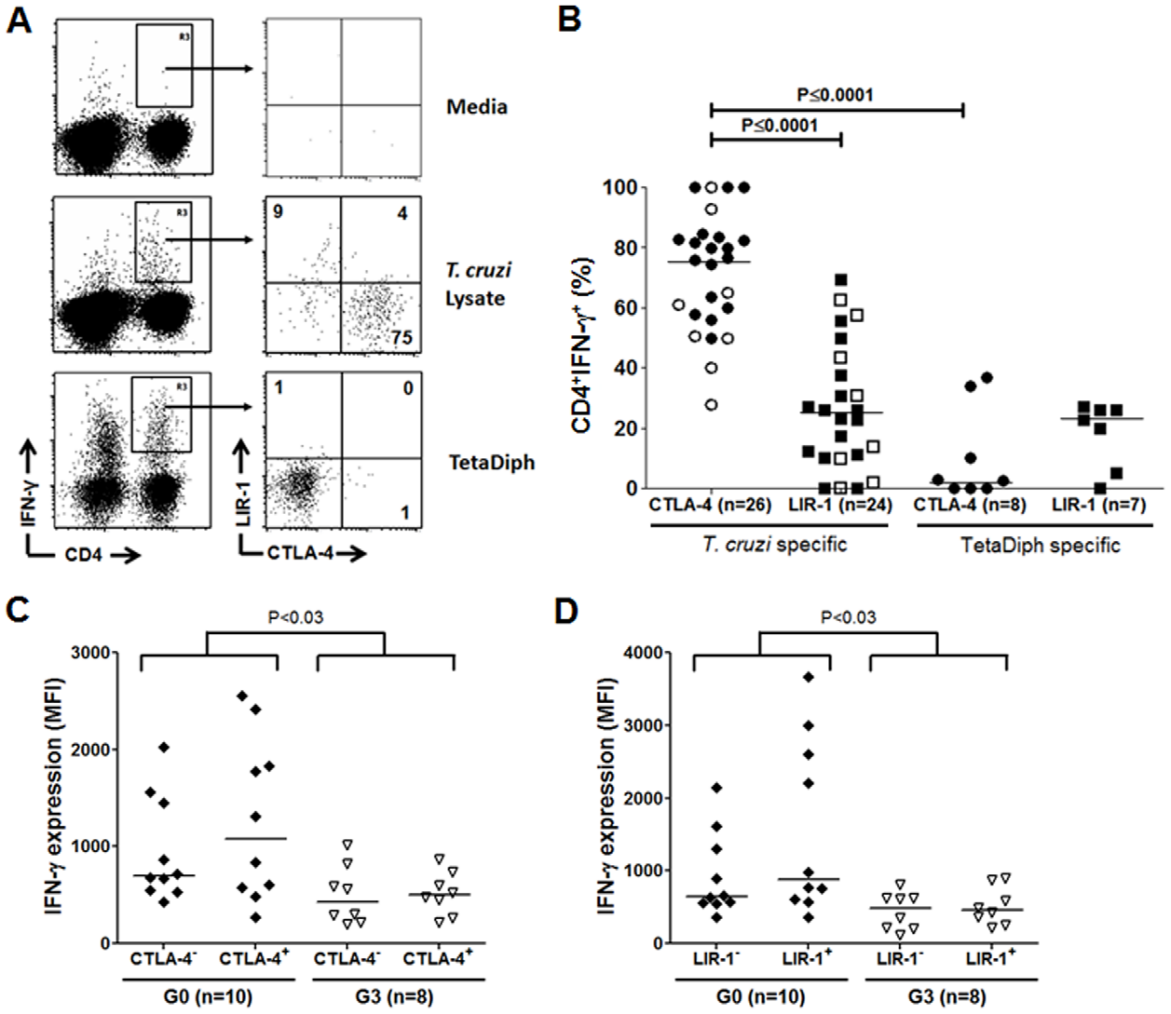
CTLA-4 and LIR-1 are expressed by *T. cruzi* antigen-responsive CD4^+^ T cells. PBMCs were stimulated for 18 hours in the presence of an amastigote *T. cruzi* lysate (middle panel), a mix of tetanus/diphtheria toxoids (low panel) or media alone (top panel). Cells were stained with anti-CD4 and anti-LIR-1 monoclonal antibodies followed by fixation and permeabilization for the intracellular staining with anti-IFN-γ and anti-CTLA-4 monoclonal antibodies. (A) Representative dot plot from a G0 *T. cruzi*-infected subject. Lymphocytes were gated by forward and side light scatter and subsequently analyzed by IFN-γ vs. CD4. The right graphs show CTLA-4 and LIR-1 staining of R3 gated cells. The figures indicate the percentage of CD4^+^IFN-γ^+^CTLA-4^+^ (lower right quadrant), CD4^+^IFN-γ^+^LIR-1^+^ (upper left quadrant), and double CD4^+^IFN-γ^+^CTLA-4^+^ LIR-1^+^ T cells (upper right quadrant). (B) Frequencies of CTLA-4^+^ and LIR-1^+^ T cells in the CD4^+^IFN-γ^+^ T cell compartment from 18 chronically infected subjects without cardiac symptoms (closed symbols, n = 10) or with severe cardiomyopathy (open symbols, n = 8). Median values are indicated by the horizontal lines. (C) MFI of IFN-γ on CTLA-4^+^ and CTLA-4^−^ CD4^+^ T cells specific for *T. cruzi* in asymptomatic and symptomatic *T. cruzi*-infected subjects. (D) MFI of IFN-γ on LIR-1^+^ and LIR-1^−^ CD4^+^ T cells specific for *T. cruzi* in asymptomatic and symptomatic *T. cruzi*-infected subjects.

We have previously shown that increased severity of chronic Chagas disease in humans was associated with impaired T cell responses specific for *T. cruzi* and with signs of exhaustion in the overall T cell compartment [29], [30]. To explore the possibility that negative regulatory pathways are involved in the poor *T. cruzi*- specific T cell responses in long-term infected subjects, we evaluated intracellular CTLA-4 expression – that represent most of the total CTLA-4 molecules synthesized [39] – and LIR-1 by CD4^+^ T cells subjected to *T. cruzi* antigens stimuli in chronically infected subjects without cardiac symptoms (Group G0) and in a group of patients with severe chagasic cardiomyopathy (Group G3). Since we had previously demonstrated that in chronically *T. cruzi*-infected humans, the frequency of T cells specific for defined *T. cruzi* epitopes [28], [31] or *T. cruzi*–derived recombinant proteins [40] is too low to be consistently detected, intracellular staining assays for IFN-γ production after stimulation with an amastigote lysate preparation were performed to evaluate the expression of CTLA-4 and LIR-1 by *T. cruzi* antigen-responsive IFN- γ-producing CD4^+^ T cells [28], [31], [41]. The majority of IFN-γ-producing CD4^+^ T cells responsive to the parasite lysate were found to express CTLA-4 but considerably lower frequencies express LIR-1 (Figure 1A middle panel, and Figure 1B), irrespective of the clinical status of the donor. Conversely, few IFN-γ-producing T cells responsive to tetanus and diphtheria toxoids expressed CTLA-4 and LIR-1 (Figure 1A low panel, and Figure 1B), showing that increased expression of these molecules in chronically-infected subjects is restricted to T cells responsive to *T. cruzi* antigens. A low co-expression between CTLA-4 and LIR-1 was observed on CD4^+^ IFN-γ^+^ T cells in response either to the parasite lysate (median percentage ± SD = 10±11, range = 2.1–37, Figure 1A, middle panel right graph) or to diphtheria toxoids (median percentage ± SD = 2.9±5.4, range = 0.7–12, Figure 1A, bottom panel right graph).

Although no significant differences in the amount of IFN-γ production per cell, as determined by MFI, were apparent between CD4^+^CTLA-4^+^ and CD4^+^CTLA-4^−^ (Figure 1C) or CD4^+^LIR-1^+^ and CD4^+^LIR-1^−^ (Figure 1D) T cells, irrespective of the clinical status, subjects with severe heart disease (G3 subjects) showed lower IFN-γ production in their *T. cruzi* antigen-responsive CD4^+^ T cells compared with those subjects without cardiac involvement (G0 subjects), (Figure 1C and Figure 1D).

**Figure 2:**
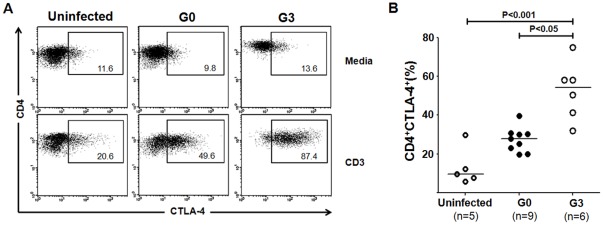
Increased frequencies of CD4^+^CTLA-4^+^ T cells following polyclonal activation with anti-CD3 antibodies. PBMCs were stimulated with anti-CD3 for 18 h or media alone. Cells were surface stained with anti-CD4 monoclonal antibody followed by fixation and permeabilization and intracellular staining with anti-CTLA-4 monoclonal antibody. Lymphocytes were gated by forward and side light scatter. From this population single color CD4 staining histogram was made and CD4^+^ T cells were selected and analyzed for CD4 vs. CTLA-4 dot plot. (A) Representative dot plots from an uninfected control, an asymptomatic subject (G0) and a patient with severe cardiomyopathy (G3). The numbers in the quadrants represent percent cells in each out of total CD4^+^ T cells. (B) The frequency of induced CD4^+^CTLA-4^+^ was calculated by subtracting the percentage of CD4^+^CTLA-4^+^ T cells in unstimulated cultures from the percentage of CD4^+^CTLA-4^+^ T cells responding to anti-CD3 stimulation. Values from individual uninfected controls, G0 or G3 subjects are depicted as separate points and median values are indicated by the horizontal lines. Kruskal-Wallis test with pairwise comparison was used to compare differences between subject groups.

With the aim of evaluating the inducible expression of CTLA-4 not only in those subjects with positive IFN-γ-responses to the parasite lysate but also in those subjects without detectable *T. cruzi* antigen-responsive CD4^+^IFN-γ^+^ T cells, PBMCs were stimulated with the pan-T cell activator, anti-CD3. CTLA-4 expression was preferentially increased among CD4^+^ T cells in G3 subjects in comparison with asymptomatic and uninfected subjects (Figures 2A and Figure 2B). Altogether, these findings show that CD4^+^ T cells from chronically *T. cruzi*-infected subjects increased CTLA-4 expression upon activation with *T. cruzi* antigens, as well as with polyclonal stimulation.

### The peripheral CD4^+^ T cell compartment in chronically *T. cruzi*-infected subjects has increased frequencies of LIR-1^+^ lymphocytes

**Figure 3:**
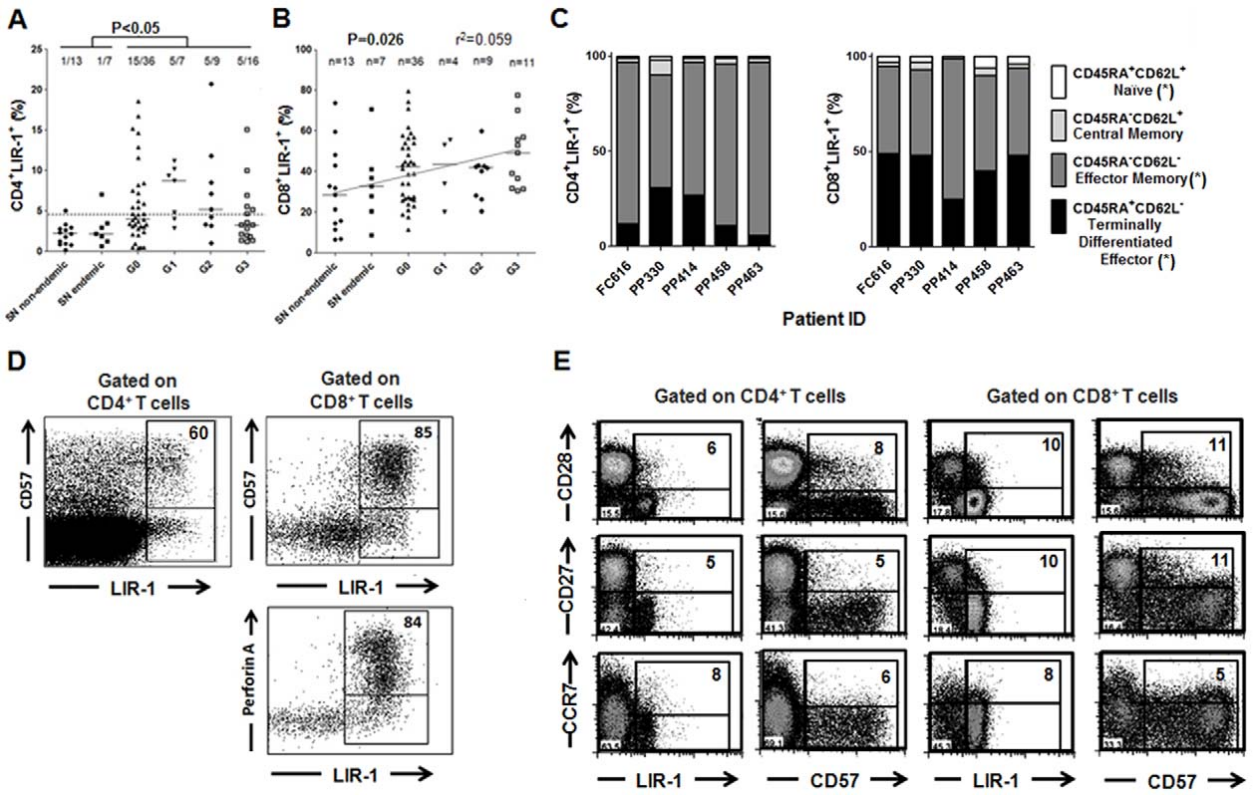
Frequencies of total LIR-1^+^ T cells in the circulation of chronically *T. cruzi*-infected subjects and uninfected controls. PBMCs were isolated by density gradient centrifugation on ficoll-hypaque and stained with anti-CD4, anti-CD8 and anti-LIR-1 monoclonal antibodies. Each point represents the percentage of CD4^+^LIR-1^+^ (A) and CD8^+^LIR-1^+^ (B) T cells in individual subjects. SN non-endemic: subjects with negative serology who had not lived in areas endemic for *T. cr*uzi infection; SN endemic: subjects with negative serology born in endemic areas; G0, G1, G2 and G3: clinical groups of chronically infected subjects as defined in Material and Methods. Median values are indicated by the horizontal lines. A cut off value for CD4^+^LIR-1^+^ T cells (dotted line) was set as the mean percentage ±2SD from uninfected subjects. The number of subjects with values above the cut off out of the total number of subjects evaluated is shown. A positive trend in the percentages of CD8^+^LIR-1^+^ T cells as disease becomes more severe is also shown. (C) Expression patterns of LIR-1 in total CD4^+^ CD8^+^ T cells. PBMCs from 4 G0 (FC616, PP414, PP458 and PP463) and 1 G1 (PP330) patients were stained with anti-CD8, anti-CD45RA, anti-CD62L and anti-LIR-1 monoclonal antibodies. The bars represent the percentages of LIR-1-expressing naïve, effector, effector memory and central memory CD4^+^ (left panel) and CD8^+^ (right panel) T cells out of total CD4^+^LIR-1^+^ or CD8^+^LIR-1^+^ T cells. (*) P<0.05 between CD8^+^ and CD4^+^ T cells for the corresponding T cell compartment expressing LIR-1. The percentages of (D) Representative perforin A and CD57 expression profiles by CD4^+^LIR-1^+^ (upper panel) or CD8^+^LIR-1^+^ (bottom panel) T cells. The percentages in the upper right quadrants show the expression of perforin A (left panel) and CD57 (right panel) by CD4^+^LIR-1^+^ or CD8^+^LIR-1^+^ T cells. Representative CD28 (top panel), CD27 (middle panel) and CCR7 (low panel) expression profiles by CD4^+^LIR-1^+^ (left panel) or CD4^+^CD57^+^ (right panel) (E), and CD8^+^LIR-1^+^ or CD8^+^CD57^+^ (F) T cells. The percentages in the upper right quadrants show the expression of the indicated molecules by CD4^+^LIR-1^+^ or CD8^+^LIR-1^+^ T and CD4^+^LIR-1^+^ or CD8^+^CD57^+^ T cells.

Previous studies from our lab had demonstrated that the overall T cell compartment in chronically *T. cruzi*-infected subjects shows evidence of significant terminal differentiation, consistent with persistent parasite stimulation [29], [30]. To determine if this pattern was also accompanied by the expression of regulatory molecules, we measured the expression of CTLA-4 and LIR-1 in total, unstimulated CD4^+^ and CD8^+^ T cells from chronically infected subjects. CD4^+^LIR-1^+^ T cells are increased in *T. cruzi* infected subjects compared with uninfected controls, either from endemic or non-endemic areas (Figure 3A). Although the levels of CD8^+^LIR-1^+^ T cells between *T. cruzi*-infected and uninfected subjects were not significant different, a positive trend as disease becomes more severe was found (Figure 3B). Conversely, CTLA-4 expression was low in the total CD4^+^ (Figure S1A) and CD8^+^ (Figure S1B) T cell compartments, regardless the clinical status of the patient.

In order to determine whether the phenotype of LIR-1^+^ T cells in chronically *T. cruzi*-infected subjects was also linked to a high differentiation status, as reported for chronic viral infections [15], we determined the expression of CD45RA (i.e. a marker of antigen experience), CD62L (i.e. adhesion molecule associated with the homing to lymph nodes), Perforin (i.e. a marker of cytotoxicity exerted by effector and memory T cells), CD57 (i.e. a marker indicative of the rounds of T cell receptor events), CCR7 (i.e. a chemokine receptor associated with the homing to lymph nodes) and, CD28 and CD27 (i.e. two molecules involved in T cell costimulation) by CD4^+^LIR-1^+^ or CD8^+^LIR-1^+^ T cells from 5 to 10 patients evaluated (6 G0, 2 G1 and 2G3 subjects). LIR-1 was primarily expressed on effector memory (CD45RA^−^CD62L^−^) (Figure 3C, left panel) CD4^+^ T cells in all subjects evaluated, while LIR-1 expression by CD8^+^ T cells was almost equally distributed between effector memory and terminally differentiated effector (CD45RA^+^CD62L^−^) T cells in most subjects (Figure 3C, right panel). LIR-1 expression was low on naïve T cells but higher in CD8^+^ in comparison with CD4^+^ T cells (Figure 3C, right panel). CD4^+^LIR-1^+^ and CD8^+^LIR-1^+^ T cells appear to be antigen-experience T cells with homing to peripheral tissues, as denoted by the low expression of CCR7, CD27 and CD28 (Figure 3E). The high expression of perforin A and CD57 by CD8^+^LIR-1^+^ (Figure 3D, right panel) and CD4^+^LIR-1^+^ (Figure 3D, left panel) T cells further sustains the late differentiation status of LIR-1^+^ T, regardless the clinical status of the patients. Moreover, CD4^+^ and CD8^+^ T cells expressing LIR-1 display a similar phenotype to CD4^+^ and CD8^+^ expressing CD57 (Figure 3E).

### Changes in the levels of CD4^+^LIR-1^+^ T cells after etiological treatment with benznidazole

**Figure 4:**
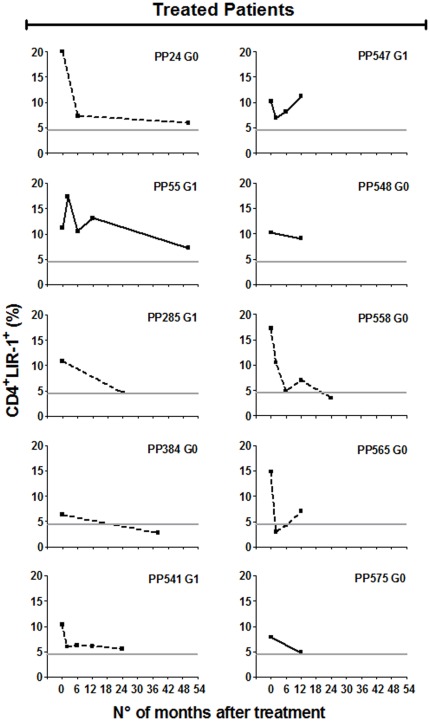
CD4^+^ T cells expressing LIR-1 following treatment with benznidazole in subjects with increased CD4^+^LIR-1^+^ T cells at baseline. PBMCs from *T. cruzi*-infected subjects were taken prior and at different time points following treatment with benznidazole and stained with anti-CD4 and anti-LIR-1 monoclonal antibodies. Plots show representative data for single subjects from 10 chronically infected subjects. Significant changes in the levels of CD4^+^LIR-1^+^ T cells, as defined in Materials and Methods are depicted with dotted lines. *Horizontal line*, cut-off CD4^+^LIR-1^+^ T cell levels in the normal range, as defined in Material and Methods.

**Figure 5:**
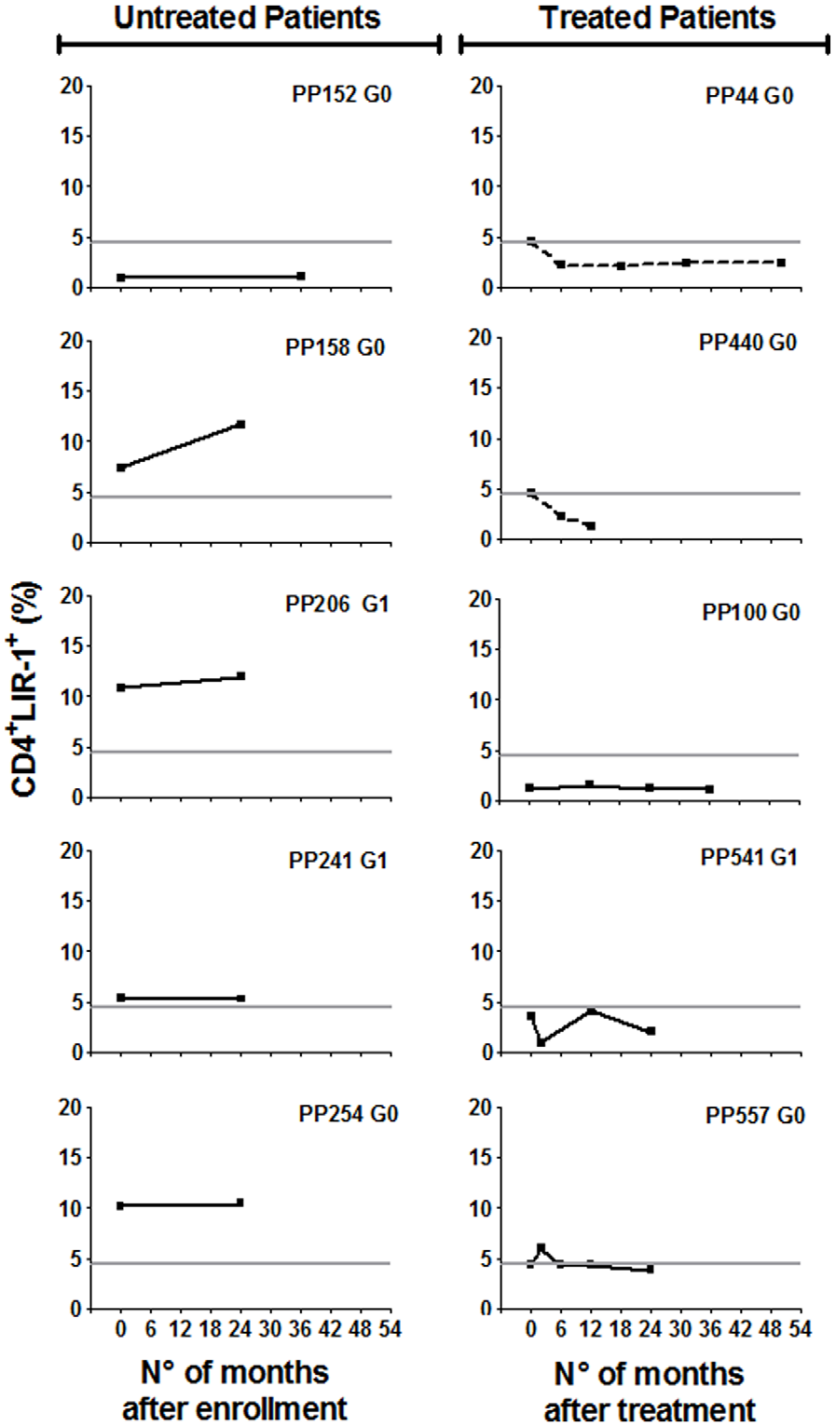
CD4^+^ T cells expressing LIR-1 following treatment with benznidazole in subjects with borderline or CD4^+^CTLA-4^+^ T cells under the cut-off value at baseline (A) and in untreated subjects after enrollment (B). PBMCs from *T. cruzi*-infected subjects were taken prior and at different time points following treatment with benznidazole or enrollment (in untreated subjects) and stained with anti-CD4 and anti-LIR-1 monoclonal antibodies. Plots show representative data for single subjects from a selected group. Significant changes in the levels of CD4^+^LIR-1^+^ T cells, as defined in Materials and Methods are depicted with dotted lines. *Horizontal line*, cut-off CD4^+^LIR-1^+^ T cell levels in the normal range, as defined in Material and Methods.

We next assessed the effect of treatment with the trypanocidal drug, benznidazole , on LIR-1 expression in total T cells. Although benznidazole treatment is not uniformly effective, we have previously shown that the majority of subjects treated by this protocol had altered T cell and declining antibody responses consistent with an efficacious outcome compatible with a decrease in parasite load [41]. Following benznidazole treatment, the frequency of total CD4^+^LIR^+^ T cells significantly decreased in 6 out of 10 (60%) *T. cruzi*-infected subjects with increased CD4^+^LIR-1^+^ T cell levels prior to treatment (i.e. PP24, PP285, PP384, PP541, PP558 and PP565) (Figure 4) in comparison with the mean changes observed overtime in untreated subjects, who displayed relatively stable CD4^+^LIR-1^+^ T cells (Figure 5, left panel). CD4^+^LIR-1^+^ T cells also decreased in 2 more subjects with borderline CD4^+^LIR-1^+^ T cell levels prior to treatment (i.e. PP44 and PP440) (Figure 5, right panel). No significant alterations in the frequency of CD4^+^LIR-1^+^ T cells following treatment with benznidazole were found in *T. cruzi*-infected subjects with baseline CD4^+^LIR-1^+^ T cells in the range of uninfected subjects (i.e. PP100, PP541 and PP557) (Figure 5, right panel). This decrease was observed as early as 2–6 months following treatment and sustained in all patients for at least 2 years after treatment (Figure 5). These findings suggest that a decrease in parasite load, eventually achieved after trypanocidal treatment, appeared to be reflected by a decline in LIR-1 expressing CD4^+^ T cells.

### Engagement of CTLA-4 and LIR-1 reduces IFN-gamma production

**Figure 6:**
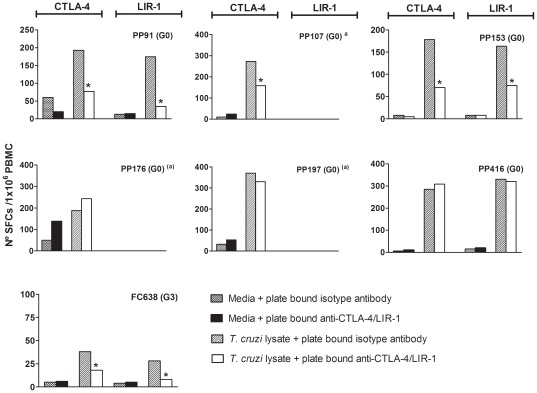
Effect of cross-linking of CTLA-4 and LIR-1 on T cell responses against *T. cruzi*-antigens. IFN-γ ELISPOT responses of PBMCs from *T. cruzi*-infected subjects stimulated with *T. cruzi* lysate or media alone were measured in the presence of a plate bound isotype control, anti-CTLA-4 or anti-LIR-1 antibodies. The data represent the mean spot number/10^6^ PBMCs for individual subjects with positive IFN-γ ELISPOT responses prior to cross-linking assays. (*) Indicates significant differences in *T. cruzi*-specific IFN-γ ELISPOT responses (SFCs in media subtracted) between previous and post cross-linking assays, as described in Material and Methods. The data represent the mean SFCs number/1×10^6^ PBMCs. ^(a)^ LIR-1 cross-linking was not performed. The clinical status of each subject is indicated between brackets.

In order to explore whether the expression of CTLA-4 in *T. cruzi*-specific T cells might have functional relevance, we measured IFN-γ ELISPOT responses to an amastigote lysate in a short-term culture of PBMCs from chronically *T. cruzi*-infected subjects after CTLA-4/LIR-1 crosslinking or blocking with monoclonal antibodies, an approach previously used to alter T cell activity in other systems [19], [42], [43]. IFN-γ responses to the lysate significantly decreased upon CTLA-4 or LIR-1 crosslinking compared with those obtained after incubation with the isotype control, in 4 out of 7 chronically infected subjects with detectable *T. cruzi* antigen-responsive IFN-γ producing T cells prior cross-linking (i.e. PP91, PP107, PP153 and FC638) (Figure 6). The levels of this receptor in patients that showed no alterations in T cell responses upon CTLA-4 cross-linking (i.e. PP176, PP197 and PP416) (Figure 6) were, in most cases, not different from the levels observed in subjects in which IFN-γ ELISPOT responses were altered following CTLA-4 cross-linking. Cross-linking had no effect on PBMCs of subjects with initial negative IFN-γ responses (data not shown). CTLA-4 blockade during IFN-γ ELISPOT assays did not result in a quantitative increase in antigen-specific CD4^+^ T cells evaluated in 8 *T. cruzi*-infected subjects with positive (Figure S2A) or negative (Figure S2B) IFN- γ ELISPOT responses specific for the lysate prior blocking.

### CTLA-4 is expressed in inflammatory heart lesions in chronically *T. cruzi*-infected subjects

Lastly, we evaluated the expression of CTLA-4 in relation to the level of inflammatory mononuclear cells in heart tissue sections (i.e. heart explants) from *T. cruzi* infected subjects with severe cardiomyopathy who had undergone heart transplantation. Since antibodies specific for LIR-1 are not available for use in formalin-fixed paraffin embedded tissues and considering the similar phenotype between LIR-1 and CD57-expressing CD4^+^ and CD8^+^ T cells (Figure 3E) , the high co-expression of these two molecules (Figure 3D), as well as a more restricted expression of CD57 by T cells compared with LIR-1 [44], the degree of cell differentiation of inflammatory mononuclear cells in heart tissues was assessed by measuring CD57 expression. From the eight heart explants from chronically *T. cruzi* infected subjects with severe cardiomyopathy analyzed, four showed severe diffuse myocarditis (mean number of infiltrating mononuclear cells ± SD = 243±160, range = 53–675), while the other 4 subjects showed mild myocarditis (mean number of infiltrating mononuclear cells ± SD = 75±46, range = 20–237).

**Figure 7:**
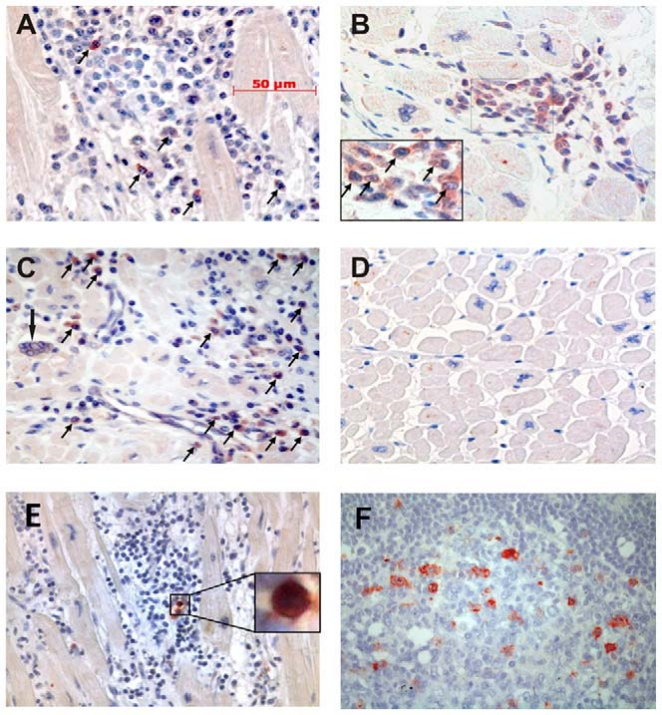
CTLA-4 and CD57 expression in the heart of chronic Chagas disease patients with severe cardiomyopathy. CTLA-4 and CD57 expression was assessed by immunohistochemistry in explanted heart tissue sections from chronic Chagas disease recipients. Two representative staining where CTLA-4 expression (arrows) was detected. Original magnification, 400× (A and B inset magnification, 1000×). Giant cell myocarditis infiltrate showing a typical giant cell (arrowhead), severe diffuse infíltrate and CTLA-4 expression (arrows). Original magnification 400× (C). No CTLA4^+^ cells were observed in idiopathic dilated cardiomyopathy heart tissues. Original magnification, 400× (D). CD57 expression in heart tissues from a *T. cruzi*-infected subject (E) and in lymph node tissues (F). Original magnification, 400×.

**Figure 8:**
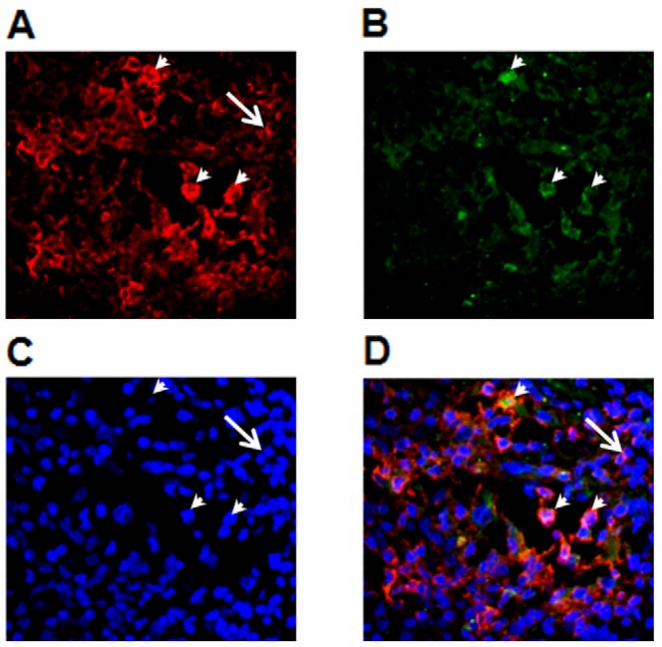
CTLA-4 is expressed by infiltrating T lymphocytes in the heart of chronic Chagas disease patients. Double immunofluorescence staining with CD3 and CTLA-4 antibodies was performed as described in Material and Methods. From total CD3-expressing T cells present in the inflammatory infiltrate (A) a small proportion showed CTLA-4 expression (B). Nuclei staining with DAPI. The arrowheads point the nuclei of CTLA4^+^ cells (C). Composite of figures A, B and C showing the double stained cells (arrowheads) and a CD3^+^CTLA-4^−^ single stained cell (large arrow)(D). Original Magnification 400×.

All 4 heart explants from chronically infected subjects with diffuse severe myocarditis showed a variable number of CTLA-4^+^ cells (average percentage of CTLA-4^+^ cells/total infiltrating cells counted = 7,6±6; range = 1–23% in 10 representative fields per patient) [Figure 7A and 7B]. Of note, the area with the highest number of CTLA4^+^ cells was observed in a section adjacent to one having amastigote nests, showing that CTLA-4 expression is increased at target tissues. No CTLA-4^+^ cells were detected in the 4 remaining cases with mild myocarditis. As expected, acute cases of giant cell myocarditis (Figure 7C) also showed intense CTLA4 expression (average percentage of CTLA-4^+^ cells/total infiltrating cells counted = 11,5±8; range = 1–24% in 10 representative fields per patient), whereas CTLA-4^+^ cells were not detected in idiopathic dilated cardiomyopathy where inflammation was not apparent (Figure 7D), confirming the association between the presence of inflammation and CTLA-4 expression. In order to confirm whether CTLA-4 expressing cells were T lymphocytes, double-immunofluorescence staining with CD3 and CTLA-4 antibodies was performed. CTLA-4 expression was mainly detected in CD3^+^ T cells in areas of severe diffuse myocarditis (Figure 8A–D).

In contrast to the high expression of CTLA-4 observed in the heart of G3 subjects, CD57^+^ cells were few and scattered in all sections evaluated, regardless of the intensity of the infiltrate (average percentage of C57^+^ cells/total infiltrating cells counted = 2,3±3; range = 1–6% in 10 representative fields per patient, Figure 8E); while high frequencies of CD57^+^ cells were observed in germinal centers of lymph nodes (Figure 6F), as previously described [25].

## Discussion

The establishment of an exhausted condition in T cell responses is linked to the constitutive expression of several inhibitory receptors that might negatively regulate the function of antigen specific T cells and thus compromise pathogen control [6], [19], [45], [46]. We have previously demonstrated that the majority of subjects in the indeterminate phase (G0 in Kuschnir classification) but only a few in the most severe stages of the chronic infection display circulating IFN-γ^+^IL-2^−^ producing effector/effector memory CD4^+^ T cells specific for *T. cruzi* antigens [28], [31], compatible with a process of immune exhaustion.

Herein, we show that *T. cruzi*–responsive CD4^+^IFN-γ^+^ T cells in the circulation of chronically infected subjects mainly express CTLA-4, while the expression of LIR-1 was generally lower, regardless of the clinical disease status in donors. Conversely, a higher expression of CTLA-4 upon pan-activation of the peripheral T cell compartment was observed in patients with advanced heart disease. This discrepancy might be due to a higher activated status of T cells in symptomatic subjects [29], [30], that eventually allow T cells to be more ready to react to polyclonal stimulation.

CTLA-4 expression has been positively correlated with viral load but negatively correlated with CD4^+^ T cell count [19]. Likewise, CTLA-4 expression has been associated with increased disease severity in human malaria disease [47]. Upregulation of CTLA-4 expression by peripheral CD4^+^ and CD8^+^T cells from subjects in the indeterminate but not in the cardiac forms of the disease, upon exposure to autologus *T. cruzi*-infected monocytes was reported [48] ; whereas in another study increased percentages of *T. cruzi* antigen-responsive CD4^+^CD25^hi^CTLA-4^+^T cells were only observed in patients with cardiac disease [49]. CD8^+^ T cells from newborns congenitally infected with *T. cruzi* exhibited increased expression of killer-cell immunoglobulin receptors by effector and effector memory T cells [50]. As a whole, the expression of inhibitory receptors by T cells in chronically *T. cruzi*-infected subjects appears to be an indicator of failed parasite clearance. However, persistent antigen stimulation might also induce the expression of inhibitory receptors to modulate a potentially overeactive immune response.

In the experimental acute *T. cruzi*- infection in mice, an upregulation of CTLA-4 expression in lymphocytes was reported, and the blockade of the signaling pathway mediated by this receptor, *in vivo* and *in vitro* led to increased inflammation but decreased tissue parasitism [51]. It was also demonstrated that CTLA-4 blockade ameliorated the outcome of the disease and increased survival rate [52]. Likewise, the expression of another inhibitory receptor, the Programmed death cell receptor 1 (PD-1), has been shown to be increased by spleen-derived T lymphocytes [53], as well as in lymphocytes infiltrating heart tissues in response to acute *T. cruzi* infection in mice, while PD-1 blockade led to reduced tissue parasitemia but increased mortality [53].

Coexpression of CTLA-4 and the hallmark of T cell exhaustion, PD-1, by CD4^+^ T cells specific for chronic viral human infections has been also demonstrated [19], [54], particularly in association with progressive HIV disease [19]. The expression of PD-1 on T cells has been related both to their differentiation stage and their activation status, being mainly expressed on early/intermediate differentiated populations that can be further upregulated as these cells become activated [55]. Therefore, it can be speculated that PD-1 might be expressed along with CTLA-4, rather than with LIR-1, by T cells in chronically *T. cruzi*-infected subjects.

The low CTLA-4 and LIR-1 expression by CD4^+^IFN-γ-producing T cells specific for a cleared protein vaccine as tetanus/diphtheria, in contrast to the high CTLA-4 and or LIR-1 expression by *T. cruzi* antigen-responsive CD4^+^ T cells, supports the conclusion that chronic antigen stimulation with *T. cruzi* drives CTLA-4 and LIR-1 expression on T cells. However, the low co-expression between CTLA-4 and LIR-1 by CD4^+^IFN-γ^+^ T cells might be explained by a different regulation of these two molecules; while CTLA-4 expression was mainly associated with T cell activation [56], LIR-1 appears to reflect the extent of T cell differentiation, as indicated by its distinct expression on highly differentiated T cells, as shown in the present and previous studies [15]. Since we have previously shown that IFN-γ-producing CD4^+^ [29] and CD8^+^ [30] T cells specific for *T. cruzi* are enriched in early differentiated CD28^+^ CD27^+^CD57^−^ T cells, it is reasonable to think that CD4^+^IFN-γ^+^CTLA-4^+^ T cells are mainly effector T cells recently recruited from the naïve pool, while highly differentiated CD4^+^IFN-γ^+^LIR-1^+^ T cells belong to the effector memory pool, which would constitute a smaller T cell population in chronically *T. cruzi* infected subjects.

CTLA-4 and LIR-1 engagement resulted in decreased IFN-γ production by *T. cruzi* –stimulated PBMC from chronically *T. cruzi*-infected subjects indicating that parasite–specific T cell responses might be regulated by inhibitory pathways. Upon binding to HLA class I molecules [26], [27], CD4^+^ T cells expressing LIR-1 might be inhibited, thus affecting macrophage activation and CD8^+^ T cell responses which are crucial to control *Trypanosoma cruzi* infection [57]–[59]. However, few studies have also reported that LIR-1 might participate in activation rather than in the inhibition of T cell responses [60], [61]. Conversely, CTLA-4 blocking in short-term culture assays had no measurable effect on *T. cruzi*-specific IFN-γ-secreting cells. Several rounds of proliferation under CTLA-4 blocking conditions may be required to recover cytokine production, as previously reported in HIV infection [19].

The rise in total CD4^+^LIR-1^+^ and CD8^+^ T cells in the circulation of chronically infected subjects is in agreement with the previously reported high differentiated stages of total T cells, likely induced by bystander activation [29], [30]. The decline in the levels of total CD4^+^LIR-1^+^ T cells after treatment with benznidazole along with decreases in *T. cruzi*-specific T and B cell responses [41] is consistent with a reduction in antigen exposure and T cell differentiation. Since, decreases in CD4^+^LIR-1^+^ T cells following treatment were sustained in most patients for over two years, we disbelieve the possibility that this decrease is due to an immunomodulatory effect of benznidazole but rather to a decrease in parasite load.

There is strong evidence that *T. cruzi* drives the inflammatory reaction in chronic chagasic myocarditis [2], [62], [63] and that this reaction is effective in controlling parasite replication but nonetheless inadequate to completely clear the infection. It is worth mentioning that a significant number of CTLA-4^+^ T lymphocytes in heart tissues was observed in areas with amastigote nets and intense myocarditis but not in areas with mild myocarditis, providing a link between parasite persistence, disease severity and CTLA-4 expression. The low frequency of CD57^+^ cells, an established marker of effector memory cells [64] and differentiation with low proliferative capacity [44], [65] in heart tissues is in agreement with the generally lower number of peripheral *T. cruzi*-specific CD4^+^ T cells expressing LIR-1 and CD57 [29], in comparison with those that express CTLA-4, CD27 and CD28 [29], [30], further supporting that effector T cells recruited from the naïve pool constitutes a major T cell population at target tissues. Of note, it has been well established that these newly recruited effector T cells generated in an scenario of persistent antigen stimulation display impaired function [66], [67], which is one of the main feature of exhausted T cells.

The possible scenario that emerges from our findings is that parasite persistence after the acute infection sustains the upregulation of CTLA-4 and LIR-1 during chronicity, with a continuous recruitment of *T. cruzi*-specific short-live effector T cells expressing CTLA-4 and the presence of fewer LIR-1-expressing effector memory T cells. Early during the chronic infection, parasite specific T cells are present allowing subjects to remain asymptomatic. Later in the infection under repeated parasite antigen exposure and enduring inhibitory signals, the ability to recruit parasite specific T cells is first decreased and finally lost, generating a higher inflammatory response in order to control the parasite at target tissues which might lead to disease progression. In agreement with this notion, we have previously reported that naïve CD4^+^ T cells are diminished during the chronic phase of *T. cruzi* infection [29], [30], particularly in patients with mild or severe heart disease [29]. Moreover, it is possible that overtime the expression of other inhibitory receptors, besides CTLA-4 or LIR-1, by CD4^+^ T cells takes place. Co-expression of multiple distinct inhibitory receptors was associated with greater CD4 and CD8 T cell exhaustion and more severe chronic viral infections [68]–[70]. Although, differences in the capacity to produce IFN-γ between CTLA-4^+^/LIR-1^+^ and CTLA-4^−^/LIR-1^−^ T cells were not found, T cells with different phenotype may differ in their capacity to exert other T cell functions, like TNF-α production and cytotoxicity activity which are lost later than IL-2 but earlier than IFN-γ production [11], issues that deserve further evaluation. We have recently observed a variable frequency of *T. cruzi* antigen-responsive CD4^+^IFN-γ^+^TNF-α^+^ T cells in chronically *T. cruzi*-infected subjects (Pérez-Mazliah, Personal communication). Thus, T cell responses specific for *T .cruzi* might first be dampened in quality and magnitude followed by a deletion of parasite specific T cell clones overtime.

The upregulation of inhibitory receptors by CD4^+^ IFN-γ^+^ T cells in response to *T. cruzi* antigens and by T cells infiltrating the heart of patients with severe cardiomyopathy further demonstrates the influence of antigen persistence on the host immune system in the chronic phase and might be another factor involved in disease progression.

## Supporting Information

Figure S1 **Frequencies of total CTLA-4^+^ T cells in the circulation of chronically** ***T. cruzi*** **-infected subjects and uninfected controls.** PBMCs were isolated by density gradient centrifugation on ficoll-hypaque and stained with anti-CD4, anti-CD8 and anti-CTLA-4 monoclonal antibodies. Each point represents the percentage of CD4^+^CTLA-4^+^ (A) or CD8^+^CTLA-4^+^ (B) T cells in individual subjects. SN non-endemic: subjects with negative serology who had not lived in areas endemic for *T. cr*uzi infection; G0, G1, G2 and G3: clinical groups of chronically infected subjects as defined in Material and Methods. Median values are indicated by the horizontal lines.
(TIF)

Figure S2 **IFN-γ-producing T cells in response to** ***T. cruzi*** **antigen stimulation after CTLA-4 blockade.** IFN-γ producing cells upon stimulation with *T. cruzi* lysate or media alone in the presence of either an isotype control or anti-CTLA-4 antibodies were measured by ELISPOT in 4 subjects with positive (A) and 4 with negative (B) IFN-γ ELISPOT responses prior to blocking assays. The data represent the mean SFCs number/1×10^6^ PBMCs. The clinical status of each subject is indicated between brackets.
(TIF)
