# Supplementary figures and images for: Inhibitory Receptors Are Expressed by Trypanosoma cruzi-Specific Effector T Cells and in Hearts of Subjects with Chronic Chagas Disease
Source: PLoS One. 2012 May 4;7(5):e35966. doi: 10.1371/journal.pone.0035966 (PMC3344843; doi:10.1371/journal.pone.0035966)

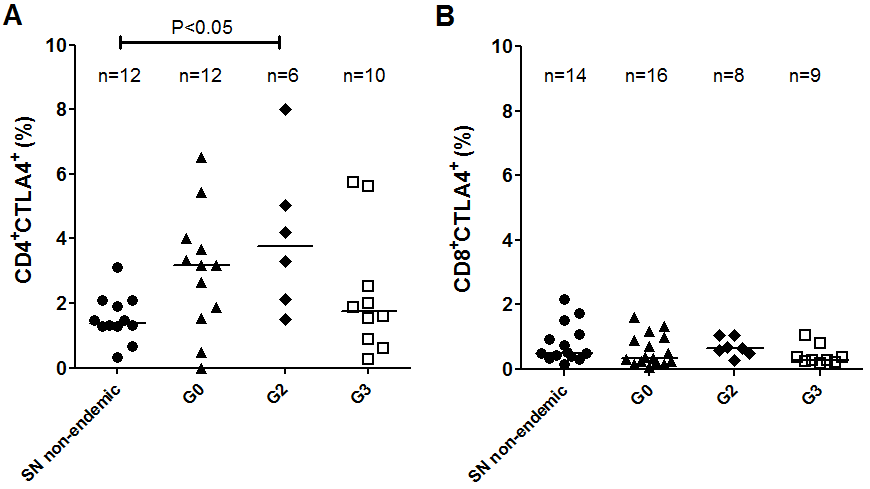

Supplement: Figure S1 — Frequencies of total CTLA-4+ T cells in the circulation of chronically T. cruzi -infected subjects and uninfected controls. PBMCs were isolated by density gradient centrifugation on ficoll-hypaque and stained with anti-CD4, anti-CD8 and anti-CTLA-4 monoclonal antibodies. Each point represents the percentage of CD4+CTLA-4+ (A) or CD8+CTLA-4+ (B) T cells in individual subjects. SN non-endemic: subjects with negative serology who had not lived in areas endemic for T. cruzi infection; G0, G1, G2 and G3: clinical groups of chronically infected subjects as defined in Material and Methods. Median values are indicated by the horizontal lines. (TIF) [file pone.0035966.s001.tif]

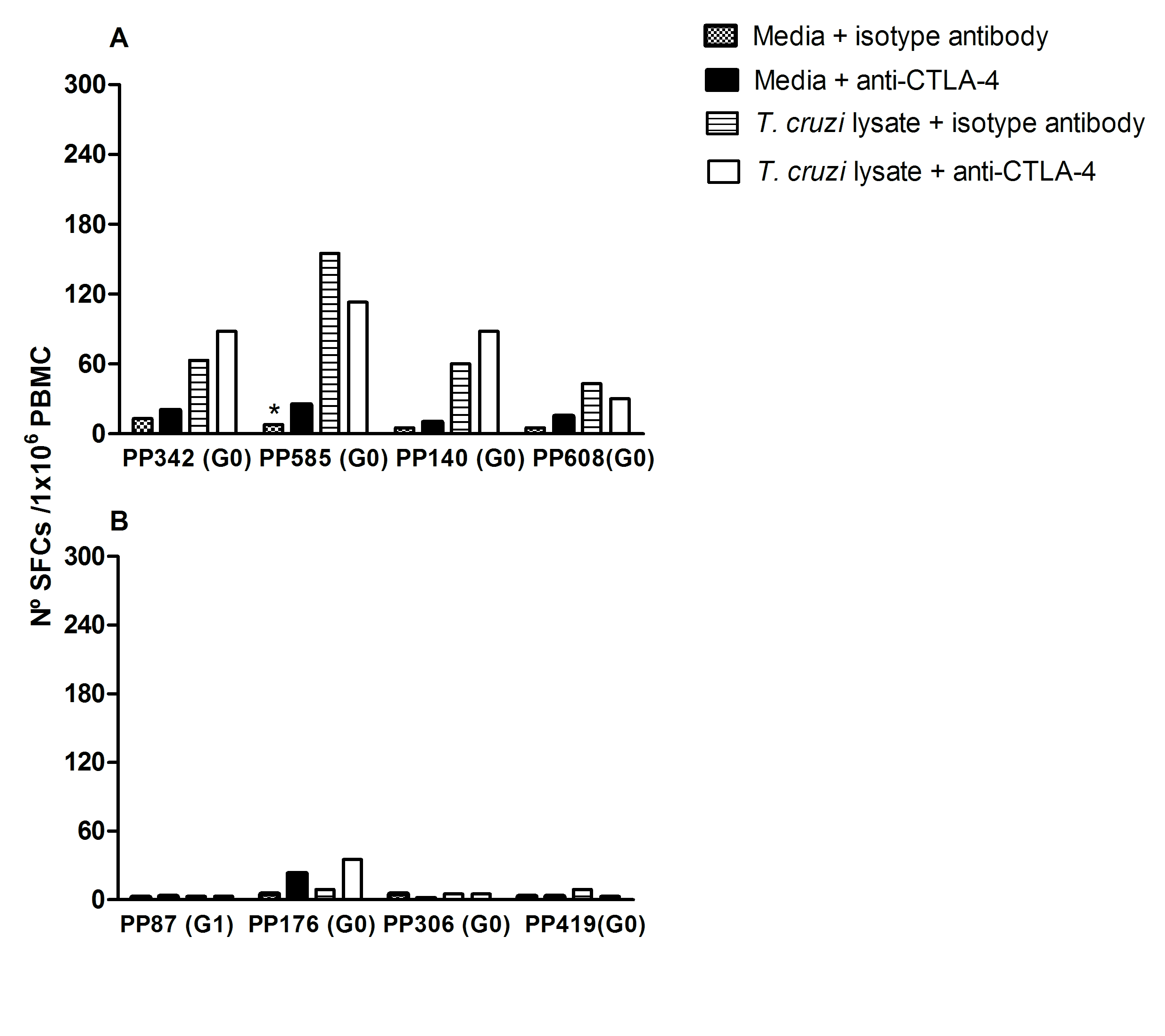

Supplement: Figure S2 — IFN-γ-producing T cells in response to T. cruzi antigen stimulation after CTLA-4 blockade. IFN-γ producing cells upon stimulation with T. cruzi lysate or media alone in the presence of either an isotype control or anti-CTLA-4 antibodies were measured by ELISPOT in 4 subjects with positive (A) and 4 with negative (B) IFN-γ ELISPOT responses prior to blocking assays. The data represent the mean SFCs number/1×106 PBMCs. The clinical status of each subject is indicated between brackets. (TIF) [file pone.0035966.s002.tif]
